# Brain tumour patients and COVID-19 vaccines: results of an international survey

**DOI:** 10.1093/noajnl/vdac063

**Published:** 2022-05-03

**Authors:** Mathew R Voisin, Kathy Oliver, Stuart Farrimond, Tess Chee, Philip O’Halloran, Martin Glas, International Brain Tumour Alliance (IBTA) Senior Advisors, Jean Arzbaecher, Carol Kruchko, Mary Ellen Maher, Chris Tse, Rosemary Cashman, Maureen Daniels, Christine Mungoshi, Sharon Lamb, Anita Granero, Mary Lovely, Jenifer Baker, Sally Payne, Gelareh Zadeh

**Affiliations:** 1 Division of Neurosurgery, Department of Surgery, University of Toronto, Toronto, Ontario, Canada; 2 International Brain Tumour Alliance (IBTA), Tadworth, UK; 3 McMaster University, Hamilton, Ontario, Canada; 4 Royal College of Surgeons in Ireland (RCSI), Dublin, Ireland; 5 Department of Neurosurgery, Queen Elizabeth Hospital, Birmingham, United Kingdom; 6 Division of Clinical Neurooncology, Dept. of Neurology and German Cancer Consortium (DKTK) Partner Site, University Hospital Essen, University Duisburg-Essen, Essen, Germany and German Innovation Alliance Cancer & Brain e.V

**Keywords:** COVID-19, brain tumour, vaccine, survey, patients

## Abstract

**Background:**

As the COVID-19 pandemic continues to unfold, the advent of multiple approved vaccines has led to a milestone in the fight against the virus. While vaccination rates and side effects are well established in the general population, these are largely unknown in patients with brain tumours. The purpose of this study was to determine if brain tumour patients and their caregivers have received a COVID-19 vaccine, and explore their thoughts and opinions on these vaccines.

**Methods:**

An anonymous 31-question online survey available in eight languages was conducted from June 30, 2021 to August 31, 2021. The survey was open to adult brain tumour patients over the age of 18 and included both categorical and open-ended questions. Descriptive statistics and modified thematic analyses were performed for all questions as appropriate.

**Results:**

A total of 965 unique surveys were completed from 42 countries. The vast majority of both brain tumour patients and their caregivers have been vaccinated against COVID-19 (84.5% and 89.9%, respectively). No patient reported serious adverse events from any vaccine. Less than 10% of patients decided against receiving a vaccination against COVID-19, with the most common reason being concerns over the safety of the vaccine. Patients wanted more specific information on how COVID-19 vaccines might impact their future brain tumour treatment.

**Conclusions:**

In conclusion, the majority of brain tumour patients and their caregivers have received COVID-19 vaccines with no major side effects. Patients want more information on how COVID-19 vaccines might directly impact their brain tumour and future management.

Funding / conflicts of interest: While no specific funding supported this manuscript and there are no real or perceived conflicts of interest in terms of this specific manuscript, the IBTA acknowledges that it does accept educational and non-directed grants for its work from a number of pharmaceutical and medical device companies. The IBTA also accepts a small number of donations from the general public and on occasion has been supported by private trusts / bequests. For more information about the IBTA's sponsorship policy and funders, please visit www.theibta.org.

Conclusions:

In conclusion, the majority of brain tumour patients and their caregivers have received COVID-19 vaccines with no major side effects. Patients want more information on how COVID-19 vaccines might directly impact their brain tumour and future management.

**Key points:**

- The majority of brain tumour patients and their caregivers have received COVID-19 vaccinations
- No major vaccination side effects were reported in this group
- Patients want more specific information on how COVID-19 vaccines might impact their future care

**Importance of the Study:**

This is the first international survey completed with the purpose of determining if adult brain tumour patients and their caregivers have been vaccinated against COVID-19 and their thoughts and opinions on the vaccines. Overall, we had close to 1000 unique responses from over 40 countries when the survey was conducted in July and August of 2021. Results demonstrated that the vast majority of brain tumour patients and their caregivers have been vaccinated against COVID-19 with no major side effects reported. Less than 10% of patients decided against vaccination, with the main concern over the safety of COVID-19 vaccines. A common theme emerged of patients wanting more specific information on how these vaccines might interact with their brain tumour directly, or indirectly impact their future brain tumour treatment.

**Introduction:**

As the global COVID-19 pandemic continues, caused by severe acute respiratory syndrome coronavirus 2 (SARS-CoV-2), there have been over 500 million confirmed cases of COVID-19, including over six million deaths as of April19, 2022.[1] With the realization that vaccines could play an essential role in increasing immunity and preventing disease, a global vaccine race commenced in early 2020 with a focus on the development, manufacturing, and distribution of vaccines.[2] The ChAdOx1 nCoV-19 vaccine (AZD1222), also known as the Oxford University / AstraZeneca vaccine (brand name Vaxzevria), was the first COVID-19 vaccine to be developed.[3] It was developed at the University of Oxford Jenner Institute in early 2020 and the university and AstraZeneca announced a global development agreement on April 30, 2020.[3] Similarly, the BNT162b2 vaccine, commonly known as the Pfizer-BioNTech vaccine or brand name Comirnaty, began development early in 2020 as well.[4] The third major vaccine, mRNA-1273 or the Moderna vaccine (brand name Spikevax), was developed in collaboration with the National Institute of Health (NIH) in the United States in February of 2020 and the first patient in their Phase 1 study was dosed on March 16, 2020.[5]

On December 2, 2020, the United Kingdom became the first country to approve a COVID-19 vaccine, the Pfizer-BioNTech vaccine, for emergency use, after being tested in a large clinical trial.[6] Similarly, the United States Food and Drug Administration (FDA) approved both Pfizer-BioNTech and the Moderna COVID-19 vaccines in December 2020 for emergency use authorization.[7] The Pfizer-BioNTech vaccine went on to be the first vaccine to receive FDA approval on August 23, 2021 for individuals ages 16 and older.[8] As of April 19, 2022, there are currently 195 vaccines in development, with 687 active vaccine trials in 75 different countries, including a majority of phase II and III trials.[9] As of April 19, 2022, the World Health Organization (WHO) has approved 38vaccines for use against COVID-19, with over 11 billion vaccine doses having already been administered worldwide.[1,9]

While extensive vaccination research has been done and is ongoing, the vaccination status, impact, and programs affecting brain tumour patients worldwide was unknown. To better understand how brain tumour patients have been prioritized and responded to the emerging role of COVID-19 vaccination utility, the International Brain Tumour Alliance (IBTA) developed an anonymous international survey that was available for completion during July and August of 2021. This is the third international survey undertaken by the IBTA during the COVID-19 pandemic. The first two surveys, completed in 2020, examined the effects of the early COVID-19 pandemic on brain tumour patients, their caregivers, and brain tumour not-for-profits and charities.[10,11] The vaccines survey focused on adult brain tumour patients and their caregivers. The purpose of the study was to determine if this population has received any COVID-19 vaccine, and explore the experiences, side effects, thoughts, and opinions of vaccination in this unique population.

**Methods:**

An anonymous online survey was developed by the International Brain Tumour Alliance (IBTA) as part of the continued work of the Society for Neuro-Oncology (SNO) COVID-19 Task Force. The 31-question survey was available in eight languages (English, French, German, Italian, Japanese, Polish, Spanish, and Urdu) and included a mixture of both categorical and qualitative, open-ended questions. Informed consent was obtained from each participant prior to survey commencement. Translation support was provided by individual members of SNO, the international brain tumour patient advocacy community, and outside translation agencies. Table 1 lists all 31 questions included in the survey. The survey was globally disseminated to over 120 brain tumour charities and not-for-profits and brain tumour healthcare professionals to distribute to their patients. Responses were collected over two months from June 30, 2021 to August 31, 2021, inclusive. The survey was open to adult brain tumour patients over the age of 18 because at the time of the survey the majority of countries had not started vaccinating individuals under the age of 18. This was prior to countries providing COVID-19 booster / third dose vaccines. The data from this survey includes only the first two vaccination doses.

Responses were grouped into the following five categories: patient demographics, COVID-19 testing and vaccination status, vaccination programs, vaccination hesitancy and side effects, and caregiver vaccination status and views. Descriptive statistics were applied for all categorical questions. Open-ended, qualitative questions were reviewed using modified thematic analysis where overarching themes are extracted from the written answers.[12] This approach uses both open coding (fragments are grouped according to shared ideas) and axial coding (dominant ideas are organized into overarching themes).[12] All text responses for all questions were independently reviewed by two researchers to ensure accurate capture of overarching themes and ideas.

## Results:

*Survey Responses and Patient Demographics*

Overall, 965 unique surveys from adult brain tumour patients were completed from a total of 42 countries. The United States had the highest number of responses (20.1%, N = 170), followed by the United Kingdom (12.4%, N = 105) and Japan (11.4%, N = 96). All 42 countries with survey responses are listed in Table 2. The median age range of all patients completing the survey was 45 to 54 years old with 11.4% (N = 96) aged 65 years or older. A total of 60% of respondents were female (N = 506).

Glioblastoma (GBM) was the most common brain tumour diagnosis at 24.6% (N = 208), followed by meningioma at 18.2% (N = 154) and astrocytoma at 12.7% (N = 107). Low-grade gliomas (grade 1 and 2) accounted for 38.7% (N = 327) of patients, while high-grade gliomas (grade 3 and 4) accounted for 43.9% (N = 371) with 22.4% (N = 189) unsure of the grade of their tumour. The majority of patients (61.4%, N = 519) were diagnosed within the past five years, 20.4% (N = 172) within the past year, while 18.5% (N = 156) received a brain tumour diagnosis over ten years ago. Epilepsy was the most common medical comorbidity reported at 15.4% (N = 130), followed by hypertension (12.5%, N = 106), and depression (11%, N = 93).

*COVID-19 Testing and Vaccination Status*

At the time of the survey, 69% (N = 534) of patients had been tested for COVID-19 for any reason, including a routine test even if they were asymptomatic. Of those 534 patients who had been tested, a total of 6.2% (N = 33) tested positive. Overall, 84.5% (N = 655) of patients had received their first dose of any COVID-19 vaccine, 82.6% (N = 554) of those who were required to obtain a second dose of a COVID-19 vaccine (excluding the Johnson & Johnson single-dose vaccine) had also received their second dose. The most common vaccine received was the Pfizer / BioNTech at 53.3% (N = 369) followed by the Astra Zeneca / Oxford University vaccine at 15.3% (N = 106) and the Moderna vaccine at 13.4% (N = 93). A total of 12.3% of patients (N = 85) responded to this question that they had not yet received any vaccine, and 2.5% (N = 17) responded that they had received a mixture of vaccines for their first and second doses. A total of 28.6% (N = 219) of patients who had received a first dose of any COVID-19 vaccine responded that they were “not at all worried” about becoming infected with COVID-19, while 6.8% (N = 52) were still “very worried” that they still might become infected. These numbers were similar for patients who had received both doses of any COVID-19 vaccine, with 27.3% (N = 207) "not at all worried" and 4.6% "very worried" about still contracting COVID-19, despite having had two doses of vaccine. The survey did not explore whether or not the reported change in treatment plan was related to having received the vaccine.

For those who received a vaccine requiring two doses, the time delay between doses was less than six weeks for 50.9% (N = 264), six weeks for 14.5% (N = 75), and twelve weeks or longer in 13.5% (N = 70). In total, 26.4% (N = 156) were on active treatment for their brain tumour when they received the COVID-19 vaccine. At time of vaccination, the most common active treatment of patient respondents included chemotherapy at (49.1%, N = 107), followed by radiation (12.4%, N = 27). A total of 4.3% (N = 22) patients had a change in treatment plan after receiving their vaccine. The vast majority of the 22 patients who reported a change in treatment plan after receiving the vaccine described treatment schedule changes to chemotherapy and radiation schedules.

*Vaccination Programs*

Of those brain tumour patients who received their first one or two COVID-19 vaccinations, 55.2% (N = 428) were prioritized by their country's government. From this question concerning vaccine prioritization, 101 patients (13%) reported they had not yet received any COVID-19 vaccine at the time of the survey. The most common reason reported in the survey for prioritization to receive the COVID-19 vaccine was a brain tumour diagnosis (30.8%, N = 227), followed by age (16.4%, N = 121), and other medical conditions (9.5%, N = 70). Free-text survey responses indicated that a more specific cause of being prioritized was that the patient was actively undergoing chemotherapy treatment. In total, 80.9% (N = 564) of patients who responded to the survey felt they had received sufficient information about the COVID-19 vaccines. Free-text responses from patients who felt they had received inadequate information included the common concern regarding the need for more information about potential COVID-19 vaccine side effects specifically on brain tumour patients and brain tumour treatments. Another common theme was the general need for more information on long-term vaccine side effects, and also specifically for brain tumour patients.

One brain tumour patient wrote: “There is little or no research on the general effectiveness/side effects on having any covid vaccine if you have an existing brain tumour. I’d like to know how the vaccines could affect my brain cancer and whether the neurological issues I experience as a result of my tumour might be affected or exacerbated by the vaccine.”

A total of 31.7% (N = 220) patients had concerns about the COVID-19 vaccination program in their home countries. The most common themes from the free-text responses to this question included vaccine distribution, prioritized groups, and vaccine safety. Some patients mentioned that their brain tumour diagnosis itself was not a reason for priority. Respondents were also frustrated about the changing / lengthening interval times between doses. If an annual COVID-19 vaccine was developed similar to the annual influenza vaccine, 85.1% (N = 596) of patients in the survey responded that they would opt to receive it. Of those who would not want to receive an annual vaccine, two major themes emerged from the free-text responses: a lack of trust in COVID-19 vaccines, and the feeling that the vaccine was unnecessary. Other responses mentioned that patients would want to re-evaluate the need on a yearly basis.

*Vaccination Hesitancy and Side Effects*

A total of 7.5% (N = 51) patients responded that they have decided not to receive any COVID-19 vaccine. A total of 84 patients addressed various concerns about COVID-19 vaccines as reasons to not receive the vaccine. The most commonly cited vaccine concern was regarding the vaccine's safety (52.4%, N = 44), followed by uncertainty about the vaccine's efficacy (21.4%, N = 18). Some patients responding to the survey stated that they felt the vaccine was unnecessary (15.5%, N = 13). Free-text responses were divided into two themes; lack of trust in the vaccine and hesitancy due to brain tumour diagnosis and/or treatment.

One patient responded: "I'm worried about potential side effects as I have recently undergone treatment for my brain tumour. I support vaccination and intend to get vaccinated once more people in my country have been vaccinated and there is more research/data about the vaccines"

Table 3 lists the reported side effects from brain tumour patients receiving any COVID-19 vaccine alongside information from the Centers for Disease Control and Prevention (CDC) collected from the general population receiving the Pfizer / BioNTech vaccine (the most common vaccine administered to brain tumor patients according to our survey).[13] The most common side effects from receiving either the first or the second dose were pain in the arm (31.2%, N = 684), followed by tiredness (18.9%, N = 414) and headache 11.1%, N = 244). A total of 257 patients (11.7%) experienced no side effects from either the first or second dose of any vaccine. In the free-text area of these questions, no patient reported any major side effect from receiving any COVID-19 vaccination. Common themes around vaccination included temporary exacerbation of patients' pre-existing neurological symptoms, including numbness, headaches, and transient weakness.

*Caregiver Vaccination Status and Views*

Overall, 37.4% (N = 258) of patients that completed the survey reported that they have a caregiver. Of those 258 patients with a caregiver, 89.9% (N = 232) of caregivers had received one or two doses of a COVID-19 vaccine at the time of this survey. A separate question asked if the brain tumour patient and their caregiver's routine has changed since their caregiver received the COVID-19 vaccine (for example, was more freedom of movement outside the home available once the caregiver had also been vaccinated). A total of 89.8% (N = 212) reported that their routine had not changed after their caregiver received the vaccine. Free-text responses from those who responded that their routine has changed (3.6%, N = 24) reported that their caregiver was now able to accompany them to medical appointments or that the brain tumour patient and their caregiver went into stores or outside of the house more often than prior to vaccination. A total of 31.6% (N = 87) had their caregiver help them complete the survey.

*Final Thoughts*

The final survey question asked if there were any other comments patients would like to pass on to those responsible for vaccination programs. The overwhelming majority of responses focused on the perceived need for brain tumour patients and their caregivers to be prioritized for vaccination. A common theme was the desire for more transparent and clear information on vaccine safety to impact on brain tumour patients and their treatments. Patients reported struggling to find clear and reliable information about COVID-19 vaccine that is specifically relevant and applicable to them.

**Discussion:**

This international survey on COVID-19 vaccinations in the brain tumour patient and caregiver population was completed over two months in July and August of 2021. During this time, the Delta variant was rapidly spreading around the globe and populations were still receiving their first or second doses of vaccinations. At the time of the survey, booster doses were not yet routinely being administered or available. In total, we received 965 unique survey responses from 42 countries. While this survey was limited to adult patients only, the overall makeup of respondents was very similar to the first global IBTA survey regarding the most common brain tumour diagnoses of GBM, meningioma, and lower-grade gliomas and medical co-morbidities.[11]

Overall, while the majority of respondents (69%) had been tested for COVID-19 at the time of the survey, only a small percentage (6%) of respondents had tested positive for COVID-19. In comparison, according to the CDC, the cumulative percent positivity in the United States for nucleic acid amplification tests (NAATs) at the time of the survey was 8.1% and was 9.5% as of February 27, 2022.[14] Our survey did not clarify what percentage of COVID-19 testing performed was symptomatic vs asymptomatic or mandatory due to a hospital or clinic visit, but there does not seem to be a large discrepancy between the number of brain tumour patients that have tested positive for COVID-19 compared to the general population.

The vast majority of both brain tumour patients and their caregivers had received one or two doses of any COVID-19 vaccine at the time of the survey (84.5% and 89.9%, respectively). The rate of vaccination in brain tumor patients and their caregivers is higher than the general population rates of vaccination at the time of the survey for both the United States (58.7%) and the United Kingdom (80.8%).[14,15] The majority of brain tumour patients and their caregivers received the Pfizer / BioNTech vaccine (53.3%) which is in keeping with CDC and National Health Service (NHS) data on United States and the United Kingdom vaccination type.[14,16] There were no major side effects reported by respondents to any of the vaccine types. Minor side effects reported by brain tumor patients including both local and systemic appeared to be notably less than those reported by the general population (Table 3). Most brain tumour patients who had a two-dose vaccine received their second dose within six weeks or less (65.4%) however approximately a quarter of brain tumour patients (26.4%) were on active treatment for their brain tumour when they received their COVID-19 vaccination, with most patients receiving chemotherapy when they were vaccinated. Recent research has highlighted that cancer patients on active treatment including chemotherapy are at a higher risk of a fatal outcome due to COVID-19 infection.[17] While guidelines surrounding chemotherapy reinforce the safety of non-live vaccines during treatment, patients may be less likely to mount an optimal immune response necessary for full immunity.[18]

The majority of brain tumour patients (55.2%) were also prioritized by their government to receive a COVID-19 vaccine, with approximately one third of respondents (30.8%) prioritized due to their brain tumour diagnosis. While our survey did not clarify whether all individuals with cancer in their countries were prioritized compared to specifically brain tumour patients only, there is an overwhelming agreement that individuals with cancer should be prioritized for COVID-19 vaccination.[17] Despite this, almost one third of respondents (31.7%) had concerns about the COVID-19 vaccination program in their country, including frustration on the changing interval timings between vaccine doses and vaccine prioritization. Some respondents mentioned that their brain tumour diagnosis itself was not prioritized, and indeed, sub-prioritization schedules for cancer patients have been described, including cancer stage, type, and other individual risk factors such as age.[19]

Overall, 7.5% of respondents had decided not to receive any COVID-19 vaccination at the time of the survey. As multiple questions asked if respondents had received the vaccine, there was some discrepancy in the number of patients that reported they had not received the vaccine due to variability in response rates for each question. Approximately 12-13% of respondents had not yet received the vaccine, depending on the question (85-101 respondents). This discrepancy may have been due to respondents being able to choose which questions of the survey to answer. Of those 85 to 101 respondents who had not yet received any vaccination, at least half of them had actively chosen not to receive the vaccine (51 respondents), and 84 respondents mentioned concerns about COVID-19 mRNA vaccines specifically as a reason not to receive the vaccine. The largest concern stated by respondents choosing not to receive the vaccine was the safety of the vaccine. Another interesting and common response for vaccine hesitancy was the unknown effects of the vaccine on patients with brain tumours. This was a common theme throughout the free-text responses.

In comparing vaccine hesitancy rates from prior studies, a systematic review of global COVID-19 vaccine hesitancy published in 2021 reported that the majority of countries included in the study had vaccine hesitancy rates of 30% or less, with the United States having one of the highest vaccine hesitancy rates of 43.1%.[20] Previously published studies on blood cancer patients and individuals with cancer or other serious comorbid conditions found that vaccine hesitancy rates amongst these populations was 17% and 18.6%, respectively.[21,22]

Overall, vaccine hesitancy rates amongst brain tumor patients were lower than both the general population and other high-risk groups, and respondents wanted more specific information on how COVID-19 vaccines might directly impact their brain tumour prognosis and treatment. Brain tumor patients also experienced no major side effects and fewer minor side effects than the general population. Respondents also largely focused on the need for brain tumour patients and their caregivers to be prioritized for vaccination and for more transparent information concerning vaccine safety and impact from healthcare professionals and the government.

**Limitations:**

Similar to the first IBTA survey carried out in early 2020 and directed to brain tumour patients (adult and pediatric) and their caregivers, this survey consisted of self-selected volunteers and therefore, there may be response bias in these results. In this survey, it was not required for survey respondents to complete every question before submitting the survey, and because of this the response rate for each question is variable. Overall, the majority of questions had a response rate of 70-80%, while other more particular questions such as those specific to patients with caregivers or those that had vaccine hesitancy returned a 30% response rate. In addition, there may have been selection bias because this survey was widely distributed through brain tumour charities and not-for-profits, and the patients and caregivers who completed the survey may not be representative of the entire population. The majority of responses came from countries including the United States, Western Europe, and Japan, and the results may not be reflective of all countries or regions. Furthermore, there is a chance that some people did not do the survey because they had yet to be vaccinated, which is also a form of selection bias. Those patients without a connection to a brain tumour charity or not-for-profit may not have been able to learn about and thereby choose to complete the survey. There may also have been challenges with informing patients from underserved and under-represented populations including ethnic minorities and low-income households about the existence and availability of the survey.

**Conclusions:**

In conclusion, the majority of brain tumour patients and their caregivers worldwide have been vaccinated against COVID-19 with no major reported vaccine side effects. Despite this, there was still a large advocacy effort to continue prioritization of COVID-19 vaccination and timely delivery of vaccinations in brain tumour patients and their caregivers. The minority of patients who chose not to receive the vaccine were concerned with the vaccine's safety and any potential direct and indirect effects the vaccine may have on brain tumours and brain tumour treatments. As we move into this current age of booster doses and novel variants, it is important to continue to research the effects of vaccines on brain tumours and patient care and advocate for these vulnerable populations most at risk of COVID-19.

Acknowledgements:

We would like to sincerely thank our survey review group consisting of members of the SNO COVID-19 Task Force and the IBTA Senior Advisors; the over 120 brain tumour charities and not-for-profits together with a wide range of healthcare professionals around the world who disseminated the survey link to brain tumour patients,, and our translators: Magda Magiera; Cristina Valencia Sanchez, MD; David Gritsch, MD; Komal Syed; Sufia Zamir; Laureline Gatellier, Gentaro Tajima, Yuko Moue, Nozomi Nonaka and colleagues (Japan Association of Medical Translation for Cancer -JAMT); and Absolute Translations Ltd (London).

**Table 1: Survey questions**

**Table 2: List of countries with survey responses**

**Table 3: Reported Side Effects from COVID-19 Vaccinations**

**Table 1: Survey questions**

| Question | Number of Responses (%) |
|---|---|
| I confirm that I am a person with a brain tumour, who is over 18 years old, and I agree to the terms and conditions stated above | 965 (100) |
| What type of brain tumour do you have? | 845 (87.6) |
| What grade of brain tumour have you been diagnosed with? (Tick all that apply if you have a mixed grade tumour) | 845 (87.6) |
| When was your tumour diagnosed? | 845 (87.6) |
| How old are you? | 845 (87.6) |
| What country do you live in? | 845 (87.6) |
| How would you describe your gender? | 844 (87.5) |
| Do you have any other medical conditions apart from your brain tumour? Please select all that apply. | 845 (87.6) |
| Have you ever had any type of COVID-19 test (because of symptoms or as a routine test even if you had no symptoms)? | 774 (80.2) |
| If you have received any COVID vaccination, were you prioritised by your country's government to receive these injections? | 775 (80.3) |
| If you were prioritised by your country's government to receive a COVID vaccine injection, on what basis was this? (Please select as many as apply) | 736 (76.3) |
| Have you received your first COVID-19 vaccination? | 775 (80.3) |
| If you have received a first COVID-19 vaccination, are you still worried that you might catch COVID-19? | 765 (79.3) |

| | |
|---|---|
| If you have received your first COVID-19 vaccination, have you also received a second COVID-19 vaccination? | 764 (79.2) |
| If you have received a second COVID-19 vaccination, are you still worried that you might catch COVID-19? | 758 (78.5) |
| If you have had two doses of a COVID-19 vaccine (ie not the Johnson & Johnson vaccine which is only one dose), what was the time gap between the first and the second dose? | 687 (71.2) |
| If you have received a COVID-19 vaccination (either a single dose or a first and second dose), which vaccine have you received? | 692 (71.7) |
| Have you decided not to have any COVID-19 vaccines? | 682 (70.7) |
| If "yes", why? | 283 (29.3) |
| At the time of receiving your first and/or second COVID-19 vaccination were you on active treatment for your brain tumour? | 696 (72.1) |
| If you were on active treatment for your brain tumour when you received your first and/or second COVID-19 vaccination, what treatment was this (please select all that apply)? | 614 (63.6) |
| If your treatment plan for a brain tumour has changed because you received a COVID-19 vaccination, please explain how this has changed. | 603 (62.5) |
| If you experienced any side effects from your first COVID-19 vaccination, please tell us what they were – you may choose as many of the below options as you wish. If you have not received a first COVID-19 vaccination, please choose "Not applicable". | 692 (71.7) |
| If you experienced any side effects from your second COVID-19 vaccination, please tell us what they were – you may choose as many of the below options as you wish. If you have not received a second COVID-19 | 687 (71.2) |

| | |
|---|---|
| vaccination, please choose "Not applicable". | |
| Do you feel that you have received enough information about COVID-19 vaccinations? | 697 (72.2) |
| Do you have any concerns about the COVID-19 vaccine programme in your country (for example distribution, equal access, timing between doses, safety, etc)? | 695 (72) |
| If an annual COVID19 vaccine was developed to guard against new COVID-19 strains or variants (much in the same way influenza vaccines are given each year) would you be prepared to receive this vaccine annually? | 700 (72.5) |
| If you have a caregiver, has your caregiver received one or both doses of the COVID-19 vaccine? | 690 (71.5) |
| If you have a caregiver, and your caregiver has received one or both doses of the COVID-19 vaccine, has this changed the way you do things together? For example, if you and your caregiver have received a COVID-19 vaccine, perhaps it's now easier for your caregiver to go with you to medical appointments. | 666 (69) |
| If you have a caregiver, has that person helped you fill in this survey? | 677 (70.2) |
| With special regard to brain tumour patients and caregivers, do you have any other comments you would like to pass on to those responsible for vaccination programmes? | 176 (18.2) |

**Table 2: List of countries with survey responses**

| Country | Number of Responses (%) |
|---|---|
| United States of America | 170 (20.1) |
| United Kingdom | 105 (12.4) |
| Japan | 96 (11.4) |
| Australia | 82 (9.7) |
| Canada | 74 (8.8) |
| Denmark | 61 (7.2) |
| Ireland | 33 (3.9) |
| Netherlands | 33 (3.9) |
| Italy | 27 (3.2) |
| Singapore | 24 (2.8) |
| New Zealand | 22 (2.6) |
| Sweden | 16 (1.9) |
| France | 15 (1.8) |
| Germany | 14 (1.7) |
| Israel | 12 (1.4) |
| India | 9 (1.1) |
| Poland | 7 (0.8) |
| Norway | 6 (0.7) |
| Zimbabwe | 5 (0.6) |
| Pakistan | 4 (0.5) |
| Austria | 3 (0.4) |
| Belgium | 3 (0.4) |

| | |
|---|---|
| China | 2 (0.2) |
| Kenya | 2 (0.2) |
| Portugal | 2 (0.2) |
| Spain | 2 (0.2) |
| Albania | 1 (0.1) |
| Anguilla | 1 (0.1) |
| Belarus | 1 (0.1) |
| Benin | 1 (0.1) |
| Bolivia (Plurinational State of) | 1 (0.1) |
| Brazil | 1 (0.1) |
| Cameroon | 1 (0.1) |
| Croatia | 1 (0.1) |
| Cyprus | 1 (0.1) |
| Hungary | 1 (0.1) |
| Romania | 1 (0.1) |
| South Africa | 1 (0.1) |
| South Sudan | 1 (0.1) |
| Uruguay | 1 (0.1) |
| Venezuela (Bolivarian Republic of) | 1 (0.1) |
| Zambia | 1 (0.1) |
| Total | 845 (100) |

## Table 3: Reported Side Effects from COVID-19 Vaccinations

| Side Effect | Number of Responses (%) | | | General Population Side Effect Rate (%) |
|---|---|---|---|---|
| | First Dose | Second Dose | First + Second Dose | First + Second Dose |
| Pain in the arm | 388 (34.3) | 296 (27.8) | 684 (31.2) | 3536 (80.5) |
| Redness in the arm | 42 (3.7) | 33 (3.1) | 75 (3.4) | 227 (5.2) |
| Swelling in the arm | 43 (3.8) | 35 (3.3) | 78 (3.6) | 264 (6.1) |
| Tiredness | 207 (18.3) | 207 (19.5) | 414 (18.9) | 2332 (53.4) |
| Headache | 117 (10.3) | 127 (11.9) | 244 (11.1) | 2044 (46.8) |
| Muscle pain | 80 (7.1) | 86 (8.1) | 166 (7.6) | 1270 (29.3) |
| Chills | 57 (5) | 61 (5.7) | 118 (5.4) | 1058 (24.6) |
| Fever | 35 (3.1) | 59 (5.6) | 94 (4.3) | 832 (19) |
| Nausea | 30 (2.7) | 34 (3.2) | 64 (2.9) | -- |
| I had no side effects | 132 (11.7) | 125 (11.8) | 257 (11.7) | -- |
| Total | 1131 (100) | 1063 (100) | 2194 (100) | 4389 (100) |
